# Flooding Control by Electrochemically Reduced Graphene Oxide Additives in Silver Catalyst Layers for CO_2_ Electrolysis

**DOI:** 10.1021/acsami.4c09095

**Published:** 2024-10-11

**Authors:** Yuming Wu, Mohamed Nazmi Idros, Desheng Feng, Wengang Huang, Thomas Burdyny, Bo Wang, Geoff Wang, Mengran Li, Thomas E. Rufford

**Affiliations:** † School of Chemical Engineering, The University of Queensland, St Lucia, Brisbane, Queensland 4072, Australia; ‡ Materials for Energy Conversion and Storage (MECS), Department of Chemical Engineering, Faculty of Applied Sciences, Delft University of Technology, van der Maasweg 9, Delft 2629 HZ, The Netherlands; § Chair of Functional Materials, Department of Materials Science & Engineering, Saarland University, Saarbrücken 66123, Germany; ∥ Department of Chemical Engineering, the University of Melbourne, Parkville, Victoria 3010, Australia; ⊥ ARC Centre of Excellence for Green Electrochemical Transformation of Carbon Dioxide, The University of Queensland, St Lucia, Brisbane, Queensland 4072, Australia

**Keywords:** CO_2_ electrolysis, graphene oxide, gas diffusion electrode, flooding, electrolyzer

## Abstract

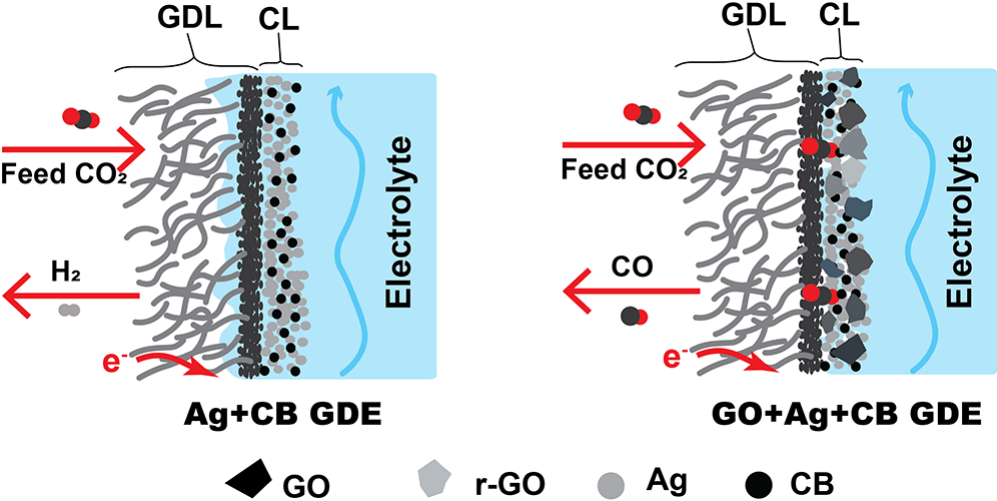

Electrolyte flooding in porous catalyst layers on gas diffusion electrodes (GDE) limits the stability and high-current performance of CO_2_ and CO electrolyzers. Here, we demonstrate the in situ electroreduction of graphene oxide (GO) to reduced graphene oxide (r-GO) within a silver catalyst layer on a carbon GDE. The r-GO introduces hydrophobicity regions in the catalyst layer that help mitigate electrolyte flooding during high current density CO_2_ electrolysis to CO. The flooding-resistant r-GO/Ag-coated GDE achieves a sustained Faradaic efficiency of CO at 94% for more than 8 h, compared to a rapid drop from 95% to 66% in an Ag-coated GDE without r-GO at 100 mA·cm^–2^. We found that GO enhances the electrochemically active surface area of the catalyst layer during CO_2_ electrolysis tests because the incorporation of GO increases the roughness of the catalyst layer. The in situ method of electrochemically reducing GO to r-GO provides a low-cost, practical approach that can be applied during standard spray-deposition procedures to develop flooding-resistant GDEs.

## Introduction

Electrochemical CO_2_ reduction, or CO_2_ electrolysis, is a promising approach to reducing carbon dioxide emissions by converting industrial CO_2_ into value-added products.^1−3^ The CO_2_ electrolysis configurations closest to industrial deployment^4,5^ use gas diffusion electrodes (GDEs) to support high surface area catalyst layers and minimize mass transfer resistance for the diffusion of CO_2_ from the gas phase to the liquid electrolyte-catalyst interface.^6^ A GDE typically comprises a carbon-based gas diffusion layer (GDL) coated with a porous catalyst layer. This catalyst layer (CL) commonly contains nanoparticles of the active catalysts, a polymer that acts as a binder and ionomer, and carbon black particles to improve the electrical conductivity of the layer.^7,8^ State-of-the-art GDE-based electrolyzers can achieve high selectivity for CO at industrially relevant current densities (>200 mA·cm^–2^).^9,10^ However, practical applications are limited by the stability of the electrolyzer performance at a high current density.

A critical factor that leads to the degradation of CO_2_ electrolyzer performance in extended high current density operation is the flooding of liquid electrolyte in the pores of the GDE.^11,12^ Liquid ingress through the electrode can block CO_2_ gas diffusion pathways to the catalyst sites, which impacts selectivity, and lead to carbonate salt precipitation across the electrode.^13^ Electrode flooding occurs over short periods as carbon surfaces in the catalyst layer and supporting GDL lose hydrophobicity under applied potential due to electrowetting phenomena.^11,14−16^ We note that polymer-based GDLs, such as those made from polytetrafluoroethylene (PTFE), are reported in lab-based studies to be flooding resistant GDEs, due to the highly hydrophobic nature of these fluorinated polymers.^10,17^ However, the low conductivity of PTFE-based GDEs^18^ has limited their scalability because nonconductive GDEs must rely on in-plane current distribution through the thin catalyst layer. As a result, PTFE and polymer-based GDLs have been limited to sizes of 1–5 cm^2^ in published CO_2_ electrolyzer studies.^17,19^ In contrast, carbon-based GDLs have already been demonstrated at commercial scales in fuel cells, hydrogen electrolyzers, and flow cell batteries.

When focusing on the catalyst layer, the wetting behavior of the catalyst surface significantly impacts the performance of the GDE within an electrolyzer.^11,12^ Several approaches have been investigated to mitigate flooding in the catalyst layer. A common approach in fuel cells and CO_2_ electrolyzers is to add hydrophobic fluoropolymer particles, such as fluoroalkyl silane^20^ or PTFE,^21,22^ to the catalyst layer. There are comprehensive studies on the effects of PTFE additives on different layers in the gas diffusion layer. For example, Kim et al.^23^ reported the effect of PTFE addition in the microporous layer (MPL) on the performance of GDE, while Shi et al.^22^ investigated the CO_2_ electrolysis on a PTFE-modified carbon fiber substrate. Nwabara et al.^24^ studied the inclusion of PTFE particles in a silver-based catalyst layer, reporting that the addition of 5 wt % PTFE for CO_2_ electrolysis results in a lower current density at all potentials compared to the other binders (e.g., Nafion and Sustainion) due to the poor electrical conductivity of PTFE.

**Figure 1:**
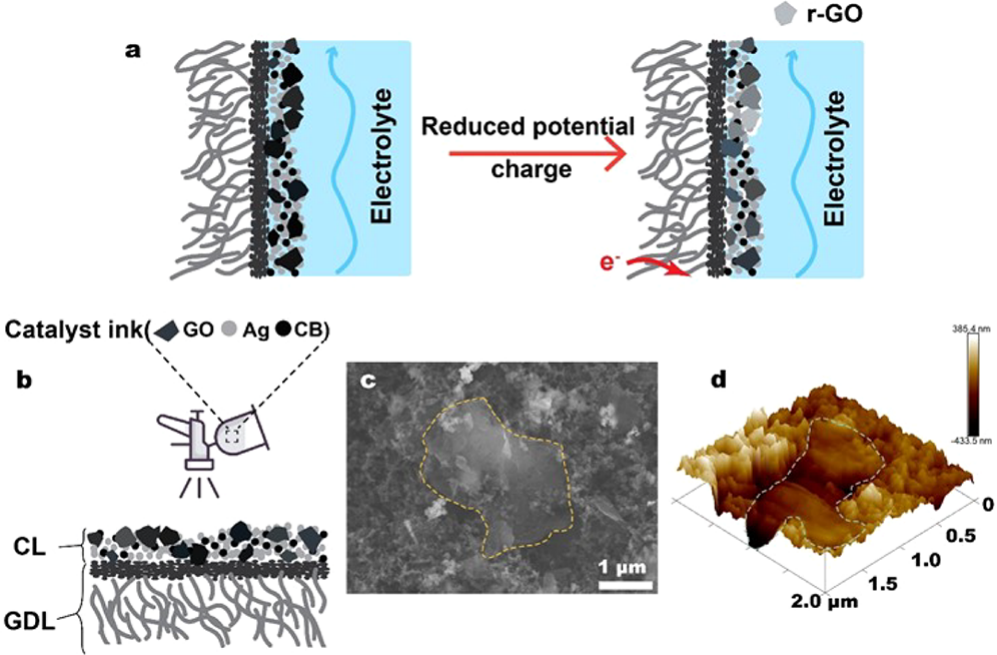
(a) Schematic of the adjustment of GDE wettability through in situ electroreduction of GO to mitigate electrolyte flooding during CO_2_ electrolysis; (b) process of spraying the catalyst ink on a commercial carbon-based GDL; (c) SEM image and (d) AFM topography of the catalyst layer on GO+Ag+CB GDE.

Nonconductive fluoropolymers can retain hydrophobicity under applied electric potentials. However, the poor electrical conductivity of PTFE and similar fluoropolymer additives can (1) reduce the electrochemically active surface available for the reactions,^21,22^ and (2) increase the cathode potential required at high-rate CO_2_ conversion, which may impact the overall energy efficiency of the process.^24^ Here, we demonstrate reduced graphene oxide (r-GO) as an additive material to the catalyst layer (Figure 1a) that is more electrically conductive than fluoropolymers but still provides a hydrophobic nature to catalyst layers for flooding resistance. Our approach builds on the work of Song et al.^25^ and Zhang et al.,^26^ who reported a reduction of GO to a hydrophobic r-GO by electrochemical methods for other applications. Studies on the performance of GDEs in fuel cells have previously reported that the addition of r-GO can improve fuel cell performance,^27^ but this has not been reported for CO_2_ electrolyzers, and in the fuel cell study, the r-GO was added to the microporous layer of the GDL, not the catalyst layer.

To confirm the electroreduction condition required in the electrolyzer, we deposited a thin film of GO flakes (Figure S1) on a glassy carbon electrode. Then we performed cyclic voltammetry and constant potential bulk electrolysis tests in an aqueous KHCO_3_ electrolyte (Figure S2a) to identify the reduction onset potentials. The CV profile of the GO thin film shows a notable peak at −1.2 V versus Ag|AgCl in the cathodic sweep from −0.2 to −1.5 V. The Raman spectra in Figure S2c following an electroreduction treatment for 10 min (in Figure S2b), indicate the degree of reduction of graphene oxide.^28,29^ The XPS spectra in Figure S2d provide consistent evidence of electrochemical reduction of the GO to r-GO with an increase in the atomic ratio of the C–C group.

We then test a hypothesis that GO particles mixed into a catalyst layer can be reduced to r-GO under CO_2_ electrolysis conditions to create a flooding-resistant catalyst layer on a GDE (Figure 1a). For high current density applications, a key advantage of r-GO compared to other hydrophobic additives, such as PTFE,^30^ fluorinated silane,^20,22^ or fluorinated ethylene propylene,^31^ is that the electrical conductivity of reduced graphene oxide flakes (6.2 × 10^2^ – 6.2 × 10^3^ S m^–1^)^32^ is many orders of magnitude higher than the conductivity of materials like PTFE (less than 6 × 10^–17^ S m^–1^).^18^ We prepared the r-GO/Ag GDE by spraying an ink of GO, Ag nanoparticles, carbon black (CB), and Nafion in 2-propanol onto a commercial gas diffusion layer (Figure 1b**)**. The compositions of the catalyst layers that we prepared are listed in Table 1. Scanning electron microscopy (SEM) in Figure 1c shows a representative GO flake in the Ag catalyst layer on the carbon GDL. The GO is evenly distributed as flakes with a lateral size of 1–2 μm (consistent with SEM and TEM of GO in Figure S1) among nanoparticles with a size distribution from 30 to 100 nm. Atomic force microscopy (AFM) results (Figure 1d) provide further information about the morphology and roughness of the GO-embedded catalyst layer.

**Table 1:** Compositions of Catalyst Inks

	GO/mg	Ag particles/mg	Carbon black/mg	Nafion/mL	Isopropanol/mL
**GO+Ag+CB**	10	50	40	0.5	2
**Ag+CB**	N/A	50	50	0.5	2
**GO+CB**	50	N/A	50	0.5	2
**GO+Ag**	50	50	N/A	0.5	2

To evaluate the performance of the GO+Ag+CB GDE, we performed CO_2_ electrolysis measurements at current densities from 100 mA·cm^–2^ using the liquid flow cell electrolyzer described in the following experimental methods and our previous work.^30^ For control experiments, we prepared a cathode electrode Ag+CB GDE with catalyst layers containing the same ionomer and silver and carbon black only (see the compositions in Table 1).

## Experimental Methods

Electroreduction of GO to r-GO: A suspension composed of 20 mg of GO (15–20 sheets, Sigma-Aldrich Pty Ltd., see Figure S1) in 4 mL of isopropyl alcohol (IPA, ≥ 99.7%, Sigma-Aldrich) was sonicated for 1 h and then dropped by a pipette onto one side of a glassy carbon plate (Carbon-Vitreous-3000C – Foil, 10 mm × 10 mm). The GO loading was 0.5 ± 0.1 mg.cm^–2^, confirmed by gravimetric analysis after evaporation of the IPA. The dried electrode was the working electrode for demonstrating GO electrocution solely.

The electroreduction measurements of GO were conducted in a three-electrode cell composed of a glass carbon plate as the cathode with active electrode area 1.5 cm^2^, Ag|AgCl (ALS Co., Ltd.) as the reference, and Pt wire as the anode. 0.1 M KHCO_3_ (≥99.5%, Sigma-Aldrich) was the electrolyte. A potentiostat charged and controlled the cell (PGSTAT204, Metrohm Autolab). Cyclic voltammetry (CV) was performed at a scan rate 50 mV/s. The electroreduction (chronoamperometry) treatment involved applying a potential of −1.2 V vs Ag|AgCl at a given time.

Electrochemical CO_2_ reduction measurements: The catalyst layer was spray coated on the MPL side of a commercial GDL (W1S1010 with MPL, 410 μm, *Fuel Cell Store*). Gas permeability and sheet resistance on the specification are 13.71 × 10^–12^ and 13 mΩ·cm^2^, respectively. The catalyst composition is included in a prepared ink. Table 1 lists the compositions of four catalyst inks. The information on materials and reagents in Table 1 is listed: GO (15–20 sheets, Sigma-Aldrich Pty Ltd., see Figure S1); Ag particles (Ag, 99.9%, 20–40 nm, Thermo Fisher Scientific); carbon black (CB, 99%, Thermo Fisher Scientific); Nafion (5 wt % perfluorinated resin, Sigma-Aldrich); isopropanol (IPA, ≥ 99.7%, Sigma-Aldrich). After sonication for 30 min, inks were sprayed onto the MPL using an airbrush (RS PRO Air Brush Kit, with 0.3 mm Tip) with a fixed airflow. The electrodes were dried at 90 °C to evaporate the IPA and water. The loadings of the catalyst layer with various components were constant (1.0 ± 0.1 mg cm^–2^), confirmed by gravimetric analysis before and after the spray coating.

CO_2_ electrolysis measurements were performed in a custom-made flow cell (Figure S9).^30^ A potentiostat charged and controlled the electrolyzer (Metrohm Autolab PGSTAT302N). The AgNP-decorated GDE served as the cathode, a 6 mm thick nickel foam (99.5%, Goodfellow Cambridge Limited) was used as the anode, and Ag|AgCl was used as the reference electrode. Nafion 117 membrane (from FuelCell Store) was adopted to separate the two chambers. The applied cathode area facing the catholyte was 1 × 1 cm^2^. A 0.1 M KHCO_3_ (≥99.5%, Sigma-Aldrich) aqueous solution as catholyte was pumped into the cathode chamber in a single pass at a flow rate of 2 mL/min. A 500 mL 1 M KOH (≥85%, pellets, Sigma-Aldrich) aqueous solution was circulated in the anode chamber at 10 mL/min. During stability tests, the anolyte was renewed every 10 h. A mass flow controller regulated the flow rate of influent CO_2_ at 60 sccm. Each current density was applied for 600 s before injecting the outgas to a gas chromatograph (Shimadzu GC-2030, more setting details in the Supporting Information) and collecting the liquid product from the effluent catholyte. The effluent catholyte was analyzed using nuclear magnetic resonance (NMR) ^1^H spectroscopy (Bruker Avance 500 high-resolution NMR; more details about product analysis and calculations are provided in the Supporting Information). Meanwhile, the electrolyte seepage rate was determined by measuring the mass of the collected seeping catholyte at a given time, as reported previously.^33^

Characterization of materials: Scanning electron microscopy (SEM) and energy-dispersive X-ray spectroscopy (EDS) were performed on a JEOL JSM-7001F instrument. Transmission electron microscopy (TEM, Hitachi HT 7700) was employed to acquire the morphology of GO flakes. Bruker Dimension ICON AFM has been used to produce AFM images of the surface of catalyst layers. The ScanAsyst mode was utilized with the ScanAsyst-Air probe, which has a spring constant of 0.4 N/m. NanoScope Analysis software was employed to analyze the AFM images and determine the average profile roughness. X-ray photoelectron spectroscopy (XPS) was conducted using a Kratos Axis Ultra XPS with a state-of-the-art Kratos Axis Ultra photoelectron spectrometer. Raman spectra were obtained by a Renishaw Raman microscope, using a laser source with a wavelength of 512 nm.

Sessile drop contact angles were measured using a goniometer designed by ourselves.^33^ We determined the contact angles by analyzing the images with ImageJ. A 1–10 μL volume pipette was used to dispense 5 ± 0.2 μL of liquid at least three different locations on each sample surface. Prior to measuring, we disassembled the electrolyzer and immersed the GDE in deionized water to thoroughly remove residual electrolyte and precipitated salts from the whole GDEs. All of the samples were dried before the contact angle measurements.

The ECSA (ECSA = A·C_dl_/loading_catalyst_) was characterized by a C_dl_-specific double-layer capacitance. C_dl_ was determined by performing ex situ CV in a single cell with three electrodes filled with 0.5 M KHCO_3_. The CV was conducted at seven scan rates (0.06, 0.08, 0.1, 0.2, 0.3, 0.4, and 0.5 V·s^–1^), as shown in Figures S10 and S11. The charging current density (taken at 0.906 V vs RHE, 0.08 V vs Ag|AgCl) was plotted versus the scan rate, where C_dl_ was calculated from the slope of this linear plot. Three ECSA measurements after each CO_2_ electrolysis measurement time were tested and their average.

## Results and Discussion

**Figure 2:**
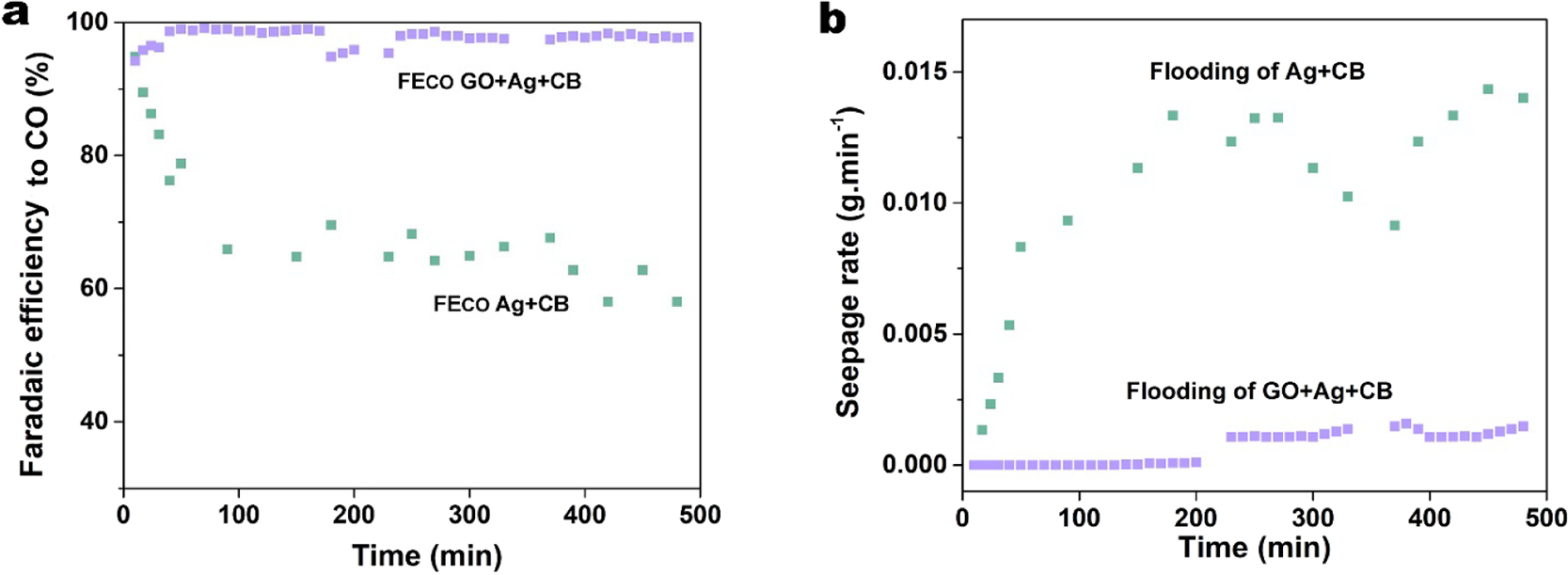
(a) Selectivity for CO and (b) real-time seepage rate for Ag+CB GDE and GO+Ag+CB GDE during an 8-h CO_2_ electrolysis test at a current density of 100 mA·cm^–2^ (data around 210 and 350 min was missing due to an adjustment of the GC autoinjection program).

Figure 2 demonstrates that the inclusion of GO in catalyst layer significantly alleviates electrolyte flooding, resulting in a stable and highly selective CO_2_ electrolysis over 8 h. In Figure 2a, the Faradaic efficiency of CO (FE_CO_) for the GO+Ag+CB GDE remains above 94% for more than 8 h at 100 mA·cm^–2^. Conversely, the performance of the Ag+CB GDE deteriorates rapidly within the first 100 min, with FE_CO_ dropping from 95% to 66%, accompanied by a noticeable rise in the electrolyte seepage rate (see Figure 2b). The seepage rate was measured to quantify the electrolyte flooding by measuring the mass of collected seeping catholyte as reported earlier.^33^ By virtue of the in situ reduction of GO in the catalyst layer under electrolyzer conditions, the exclusive stability of the GO+Ag+CB GDE is established through effective control of electrolyte flooding.

We note in Figure 2 an increase in FE_CO_ of GO+Ag+CB in the first 60 min of the stability test. This initial performance increase might be attributed to the time required for electrochemical reduction of the GO to hydrophobic r-GO. Over an extended operating time of 60 h, the stability of the GO+Ag+CB GDE was demonstrated, with FE_CO_ only declining to 77% after the prolonged operation (Figure S4). At end of the 60-h test, the seepage rate in the GO+Ag+CB GDE remains at 0.0014 g·min^–1^, similar to the rate observed during the first 8-h test (Figure 2b). We acknowledge that these stability tests are much shorter than the several thousand hours of stable operation required for industrial CO_2_ electrolyzers. Still, these results highlight the potential of using GO as a superior wet-proofing additive within GDEs in CO_2_ electrolyzers to mitigate electrolyte flooding and uphold CO_2_ diffusion pathways.

**Figure 3:**
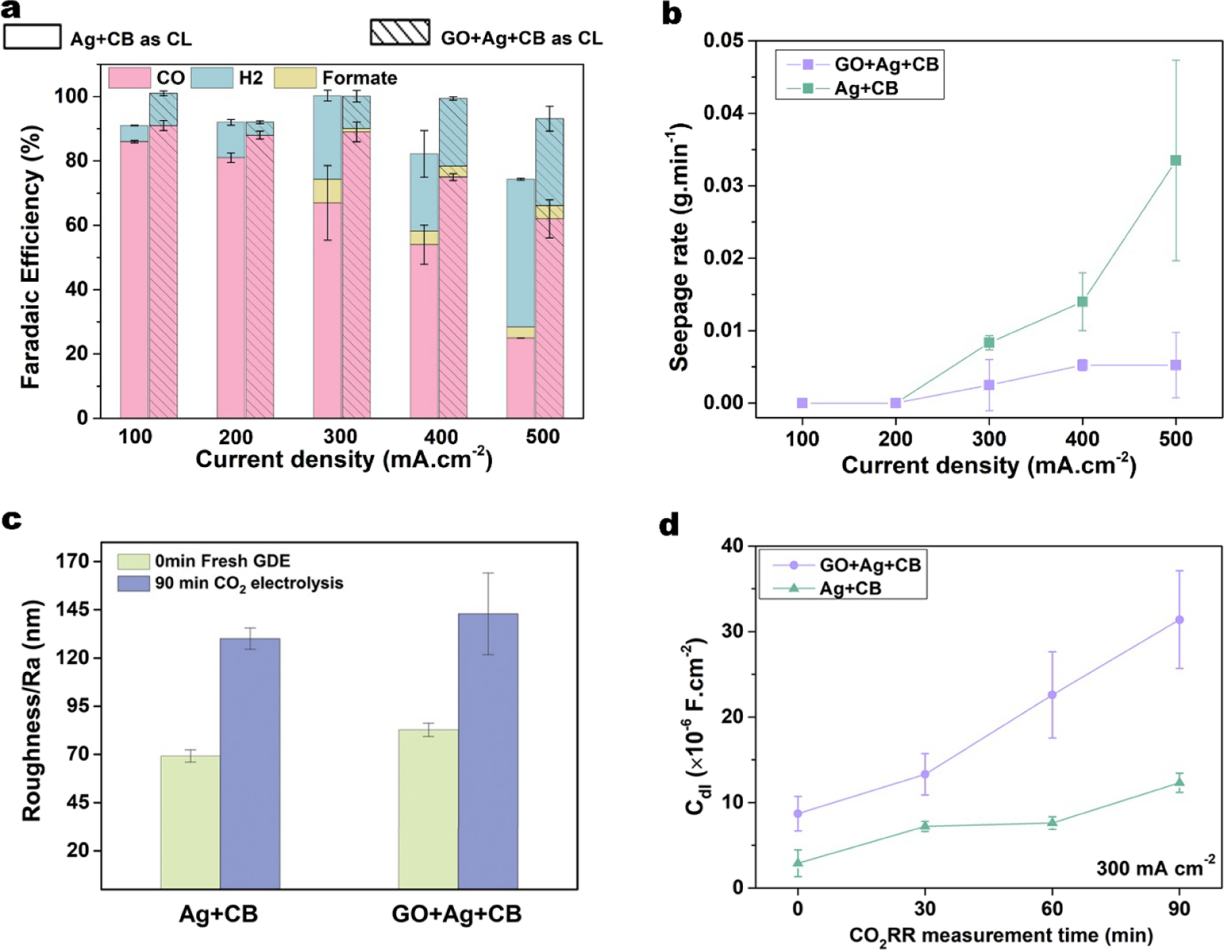
(a) Faradaic efficiency of the detectable CO, H_2_, and formate for Ag+CB and GO+Ag+CB GDEs; (b) average rate of catholyte seepage through the GDEs; (c) change of average profile roughness (Ra, obtained by the analysis of AFM image) on the two catalyst layers after 90 min of CO_2_ electrolysis at 300 mA·cm^–2^; (d) specific double layer of GDEs with the two catalyst layers before and after CO_2_ electrolysis at 300 mA·cm^–2^ for 30, 60, and 90 min. C_dl_ was calculated from the electrochemical surface area described in the Supporting Information. The error bars on FEs in (a) represent the standard deviation of three measurements of effluent gas compositions. The error bars in parts (b, c, and d) represent the standard deviation of three independent measurements on various GDEs for each measured current density and time.

To fully evaluate the performance of the GO+Ag+CB GDE, additional CO_2_ electrolysis measurements were performed at current densities from 100 to 500 mA·cm^–2^. Fresh GDEs were used at each current density to avoid any cumulative effects of electrolyte flooding or catalyst surface changes that might occur during electrochemical measurements. Figure 3a shows that the GO+Ag+CB GDE significantly impacts the distribution of cathode products at high current densities compared to the Ag+CB GDE control experiment . The primary products in each set of tests are CO and H_2_, but low concentrations of formate products are detected in the catholyte liquid at high current densities. At a current density of 100 mA·cm^–2^, the selectivity for CO for both electrodes falls within the typical range of FE_CO_ = 90–95% for Ag nanoparticle catalysts on carbon GDEs, as reported in similar experiments.^12,30,33,34^ When the current density increases to 300 mA·cm^–2^ and higher, there are significant differences in both FE_CO_ and FE_H2_ (Figure 3a) and electrolyte seepage rates (Figure 3b) between the Ag+CO electrode and the GO-embedded electrode. The seepage rate of the Ag+CB electrode increases from nearly zero at 100 mA·cm^–2^ to 0.034 ± 0.014 g·min^–1^ at 500 mA·cm^–2^, which indicates severe electrolyte flooding at high current density.^33^ When the flooded electrolyte impedes the supply of CO_2_ in the Ag+CB GDE, a drop occurs in FE_CO_ to less than 30% at 500 mA·cm^–2^ with rising H_2_ evolution and a limiting CO_2_ reduction current density of 233 mA·cm^–2^ for CO at 400 mA·cm^–2^ (Figure S6).

In contrast, the GO-embedded catalyst layer in the GO+Ag+CB GDE exhibits stable CO selectivity up to 300 mA·cm^–2^ (FE_CO_ = 88%) (Figure 3a) and then a fall to only FE_CO_ = 62% at 500 mA·cm^–2^. The observed degradation of FE_CO_ for the GO-embedded catalyst layer at current densities above 200 mA·cm^–2^ is remarkably less than that of other carbon-based GDEs. For example, Wei et al.^35^ reported a FE_CO_ fall from 95% at 200 mA·cm^–2^ to only 32.7% at 300 mA·cm^–2^ with a Ag catalyst in a similar experiment to ours. We attribute the excellent stability of the GO+Ag+CB electrode to the wetting resistance of GO reduced to r-GO in the catalyst layer under electrolyzer conditions. This conclusion is supported by the very low seepage rate in the GO+Ag+CB electrode, which remains less than 0.005 ± 0.005 g·min^–1^ at 500 mA·cm^–2^ (seven times lower seepage rate than in the Ag+CB GDE).

We note that the sum of FEs for the CO, H_2_, and formate products is greater than 90% for both electrodes and 100, 200, and 300 mA·cm^–2^, which is within the typical uncertainty of these measurements. However, we observed a more significant gap in the electron balance (Figure 3a) at current densities higher than 300 mA·cm^–2^. This experimental observation is due to the H_2_ bubbles pumped out with catholyte.^33^ Such imperfection in the electrolyzer is related to the imbalanced gas/liquid pressures across the GDE. The phenomenon of less than 100% FE is a common issue for other studies.^36,37^

To investigate the mechanism of the overall enhanced performance of CO_2_ electrolysis, we performed a series of CO_2_ electrolysis experiments with the GO+Ag+CB and Ag+CB GDEs at different electrolysis time durations. We measured surface roughness of the catalyst layer by AFM (Figures 3c and S7) and electrochemically active surface area (ECSA) (Figure 3d) of the GDE before and after the CO_2_ electrolysis at 300 mA·cm^–2^ for various time durations. Figure 3c shows that after CO_2_ electrolysis, the surface roughness of both catalyst layers is almost double their initial roughness, which may be due to the surface restructuring of the silver during electrolysis.^38^Figure 3d compares the double-layer capacitances (C_dl_) of the GO+Ag+CB and Ag+CB electrodes before and after electrolysis. The double-layer capacitance is a function of ECSA of the electrode, providing information on charge capacity and kinetics.^39^ The addition of GO prior to CO_2_ electrolysis in Figure 3d enhances the ECSA, possibly due to the more hydrophilic nature of GO compared to carbon black (as shown in Figure S3). GO’s hydrophilic properties on the GO+Ag+CB GDE increase the surface area exposed to the electrolyte during ECSA measurements. After CO_2_ electrolysis at 300 mA·cm^–2^, the double-layer capacitances of both types of electrodes increases. The similar increasing trend on GDE has been observed by other researchers.^34^ The escalating ECSA may be caused by the increased surface roughness observed in Figure 3c. This trend aligns with the findings of Jiang et al.^40^ who noted that a rising ECSA trend is associated with a rougher electrode surface resulting from plasma pretreatment. This surface roughness and ECSA results suggest that the GO-embedded catalyst layer proves to have a more active surface area with increased time, which is consistent with the seepage and FE_CO_ results presented in Figure 3a and b.

**Figure 4:**
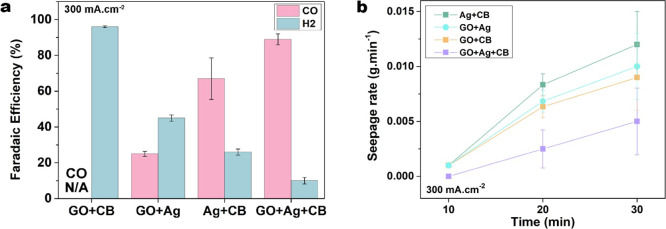
(a) Selectivity and (b) seepage rate of GDEs with different components in the catalyst layer charged at 300 mA·cm^–2^ (see Table 1 for the details of composition). The error bars on FEs in (a) represent the standard deviation of three measurements of effluent gas compositions. The error bars on seepage rates in (b) represent the standard deviation of three independent measurements on different GDEs for each measured current density.

To confirm the role of GO and r-GO in the catalyst layer, we evaluated two more GDEs with different catalyst layer formulations: (i) GO+Ag without carbon black and (ii) a control electrode of GO+CB without the Ag nanoparticles, while the same ionomer is contained in each catalyst layer, as provided in Table 1. As expected,^41,42^ we did not detect any CO_2_ reduction products in the GO+CB experiment without silver on the cathode (Figure 4a). Hence, GO/r-GO is not able to provide active sites for CO_2_ reduction, despite the inclusion of GO effectively alleviate the electrolyte flooding throughout the CO2 electrolysis (see Figure 4b). The performance in Figure 4a further demonstrates that GO flakes cannot entirely replace carbon black because GO+Ag only achieved FE_CO_ 25% at 300 mA·cm^–2^. The role of CB in the catalyst layer has been reported as a suitable dispersant for the catalyst materials by Zheng et al.^43^ We also observed the well-dispersed silver particles surrounded by CB (see Figure S8a), while the SEM images in Figure S8b show that Ag nanoparticles tend to agglomerate around the GO flakes in GO+Ag GDE. Further, the layer-by-layer GO stacking structure in the GO+Ag electrodes may inhibit through-plane gas diffusion to the catalyst layers.^44^

Meanwhile, the seepage rate of the GO+Ag GDE does not decrease as expected with the transition from GO to r-GO. The higher seepage rate compared to the GO+Ag+CB GDE might be due to the increased potential at the GO+Ag GDE, as shown in Figure S5. An increase in electrode potential can result in stronger electrowetting,^45^ where the liquid spreads more easily across the solid surface under a higher applied electric field.^11^ This enhanced electrowetting significantly contributes to electrolyte flooding in the GO+Ag GDE.

These findings suggest that there is likely an optimal ratio of GO to CB in the catalyst layer to balance the wettability and porosity effects. Optimization of the catalyst ink formulation is beyond the focus of this paper but could be an essential topic to explore in detail for improved fabrication of cathodes for CO_2_ electrolyzers.

## Conclusions

In summary, we exploited the hydrophilicity to hydrophobicity transition between GO and r-GO through the in situ electroreduction of GO on GDE in a CO_2_ electrolyzer. This reduction helps mitigate electrolyte flooding and maintain diffusion paths for CO_2_ in the GDE. The CO_2_ electrolysis measurements show that FE_CO_ maintains at 89% at 300 mA·cm^–2^. At the same time, the C_dl_ of the GDE increases with the incorporation of GO, thanks to the increased roughness of the CL surface compared to a control GDE without GO. Owing to these advantages, the GO-incorporated GDE could persistently produce CO above 94% FE for up to 8 h at 100 mA·cm^–2^. We showcase the effectiveness of incorporating GO as an alternative strategy for future catalyst layer design in large-scale CO_2_ electrolysis. Furthermore, we envisage a similar chemical transformation-induced wet-proofing effect of other 2D materials such as MoS_2_^46^ and MXene.^47^

## Supporting Information Available

Methodologies of measurement and analysis of electrochemical products; morphology of received GO and GDEs; electroreduction treatments on the received GO and related Reman and XPS characterizations; cathode potentials during the CO_2_ electrolysis; AFM topography of GDEs; NMR spectrum of collected effluent catholyte; CV curves for ECSA measurements; Faradaic efficiency for CO and H_2_ for the GO-embedded GDE in 60 h stability test; images of contact angles change after CO_2_ electrolysis (PDF)
