# Supplementary material for: Flooding Control by Electrochemically Reduced Graphene Oxide Additives in Silver Catalyst Layers for CO2 Electrolysis
Source: ACS Appl Mater Interfaces. 2024 Oct 11;16(42):56967–74. doi: 10.1021/acsami.4c09095 (PMC11505894; doi:10.1021/acsami.4c09095)
Supplement: Supplementary file 1 — am4c09095_si_001.pdf [file am4c09095_si_001.pdf]

Supporting Information

Flooding control by electrochemically reduced graphene oxide additive in silver catalyst layer for CO_2 electrolysis

Yuming Wu[a], Mohamed Nazmi Idros[a], Desheng Feng[a], Wengang Huang[a], Thomas Burdyny[b], Bo Wang[c], Geoff Wang[a], Mengran Li[d,] and Thomas E. Rufford[a,e,*]*

[a] School of Chemical Engineering, The University of Queensland, St Lucia 4072, Australia

[b] Materials for Energy Conversion and Storage (MECS), Department of Chemical Engineering, Faculty of Applied Sciences, Delft University of Technology, van der Maasweg 9, 2629 HZ Delft, The Netherlands

[c] Chair of Functional Materials, Department of Materials Science & Engineering, Saarland University, Saarbrücken, 66123, Germany

[d] Department of Chemical Engineering, the University of Melbourne, Parkville, 3010, Victoria, Australia

[e] ARC Centre of Excellence for Green Electrochemical Transformation of Carbon Dioxide, The University of Queensland, Brisbane, St Lucia, QLD 4072, Australia

Corresponding Author: aaron.li1@unimelb.edu.au (M Li); t.rufford@uq.edu.au (T. E. Rufford)

Products analysis

The compositions of gas products were analyzed with a Shimadzu GC-2030 gas chromatograph with a ShinCarbon packed column (ST 80/100, 2 mm ID, 1/8 OD Silco, Restek), including a flame ionization detector (FID) and a thermal conductivity detector (TCD). Argon (Ar, 99.999%, BOC Australia) was used as the carrier gases for the TCD. Hydrogen (H_2, 99.999%, BOC Australia) and zero grade air was used for the FID. The GC was calibrated using a standard sample gas of 1.01% H_2 and 1.01% CO balanced with Ar. Gas volumes were obtained from the output peak areas by calibration curves obtained by GC.

The Faradaic efficiencies (FE_i) of CO and H_2 were calculated by:

$$FE_i = \frac{p \times v \times c_i \times F \times N_i}{R \times T \times j} \times 100\% \qquad \text{Eq. 1}$$

where p is 101.31 kPa, v is the flow rate of effluent gas measured using a volume flowrate mater (G6691A, Agilent), c_i is the concentration of the gas product species i measured by the GC, F is the Faraday constant (96,485 C mol^{-1}), $N_i = 2$ is the number of electrons transfer for one mole CO_2 electrolysis product for both H_2 and CO, R is the gas constant (8.31446 $J \cdot K^{-1} \cdot mol^{-1}$), T is the temperature of CO_2 electrolysis reactor, and j is the total current recorded by the potentiostat.

We collected the effluent catholyte and then analyzed these samples using nuclear magnetic resonance (NMR) ^1H spectroscopy (Bruker Avance 500 high-resolution NMR). Liquid samples were prepared for the NMR analysis by mixing 400 uL of the effluent liquid from the cathode

chamber in a 200 uL mixture of heavy water (D_2O, 99.9 atom% D, Sigma Aldrich) and 0.05 vol% dimethyl sulfoxide (DMSO, ≥ 99%, Sigma Aldrich) that acts as the internal standard. Then the concentrations of liquid products were determined from the mass ratio between liquid products and DMSO. Finally, the FE of liquid (FE_l) products were calculated by the following equation:

$$FE_l = \frac{c_l \times v_{catholyte} \times F \times N_i}{j} \times 100\% \qquad \text{Eq. 2}$$

where c_l is the concentration of the liquid product, $v_{catholyte}$ is the flow rate of catholyte), and the other variables are defined the same as in Eq. 1. A representative NMR spectrum of collected effluent catholyte is shown in **Figure S13**.

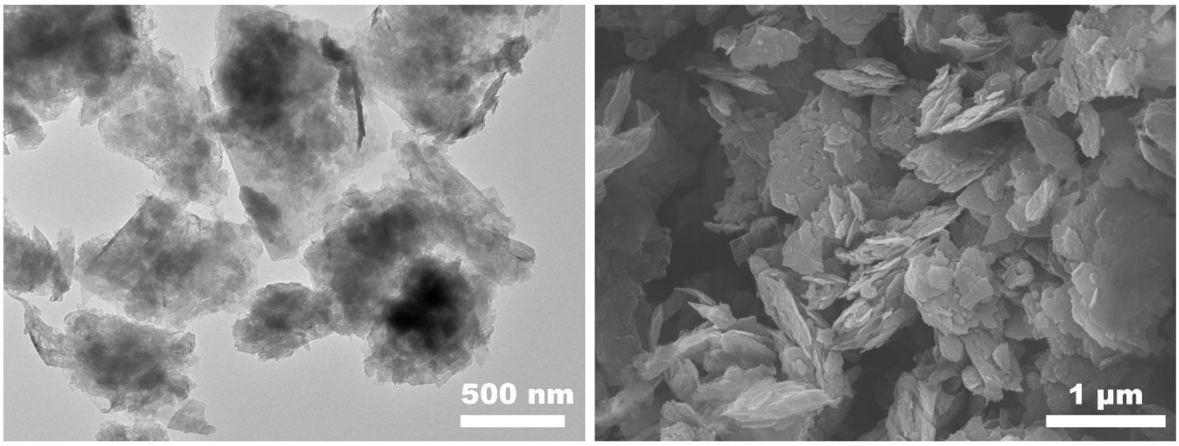

Figure S-1 Morphology of received GO: TEM image (left) and SEM image (right)

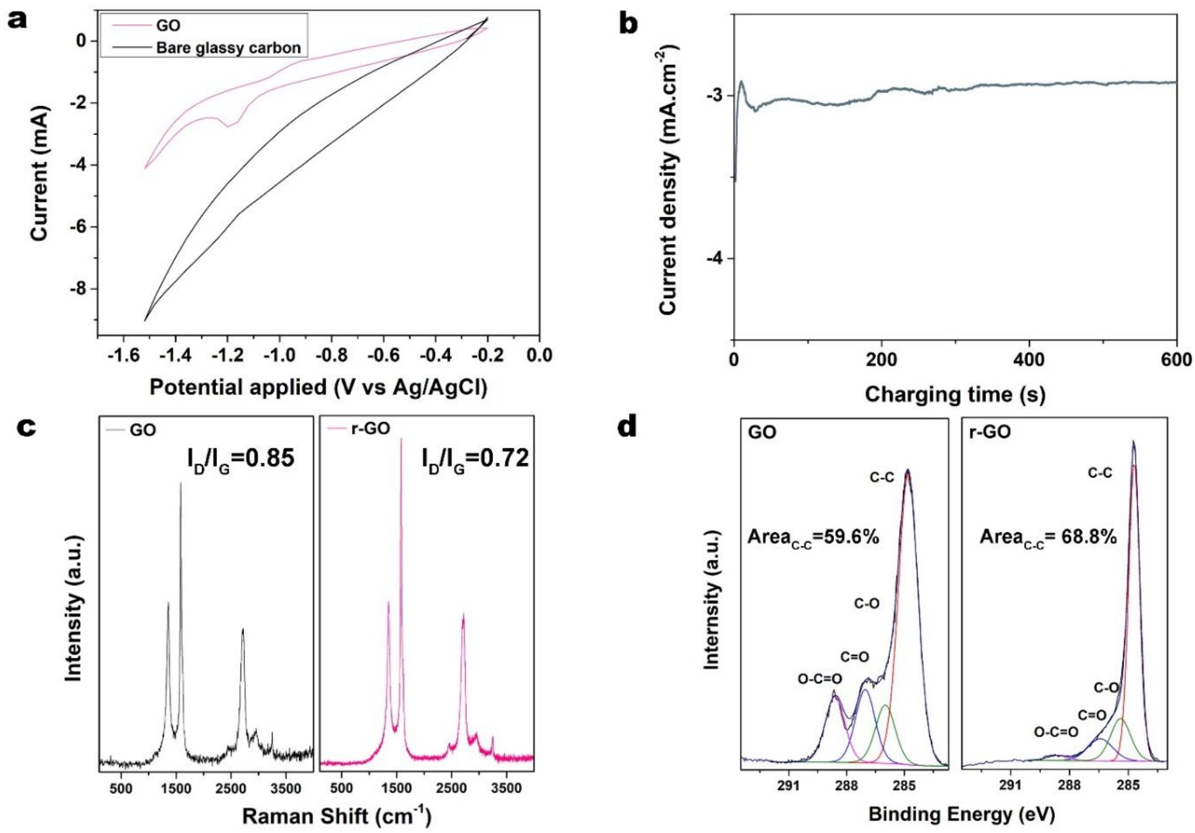

Figure S-2 (a) Cyclic voltammogram (CV) of GO at a scan rate of 50 mV/s on a glassy carbon electrode immersed in a three-electrode cell with aqueous 0.1 M $KHCO_3$ and bare glassy carbon as a control measurement; (b) Current density of GO on glassy carbon depend on time charged by chronoamperometry mode (-1.2 V vs Ag/AgCl) 0.1 M $KHCO_3$ for the electroreduction of GO to r-GO; (c) Raman spectra (laser wavelength: 512 nm) and (d) C1s region of XPS spectra of GO samples before (left) and after (right) electroreduction treatment for 30 minutes.

As reported by Ramesha et al.[1] (in 0.1 M KNO_3) and Shao et al. [2] (in 0.1 M Na_2SO_4), we conduct a cyclic voltammogram (CV) in a three-electrode cell following the methods. The CV profile of the GO thin film (**Figure S-2a**) shows a notable peak at -1.2 V versus Ag|AgCl in the cathodic sweep from -0.2 V to -1.5 V. Additionally, we performed a 10-minute cathodic charging of a GO film on a glassy carbon at a constant potential of -1.2 V versus Ag|AgCl (**Figure S-2b**). The Raman spectra in **Figure S-2c** show that after 10 minutes of the treatment at -1.2 V versus Ag|AgCl, the intensity ratio (I_D/I_G) between D-band and G-band in the GO film decreased from I_D/I_G = 0.85 to I_D/I_G = 0.72. The shift in I_D/I_G can indicate the degree of reduction of graphene

oxide [1, 3], and thus, the Raman spectra provide evidence that GO can be reduced to r-GO at -1.2 V versus Ag|AgCl in 0.1 M $KHCO_3$ electrolyte. The XPS spectra in **Figure S-2d** provide consistent evidence of electrochemical reduction of the GO to r-GO with an increase in atomic ratio of the C-C group (the deconvoluted peaks at approximately 284.8 eV) from 59.6 % in GO to 68.8 % in r-GO after 10 minutes of electrochemical conditioning.

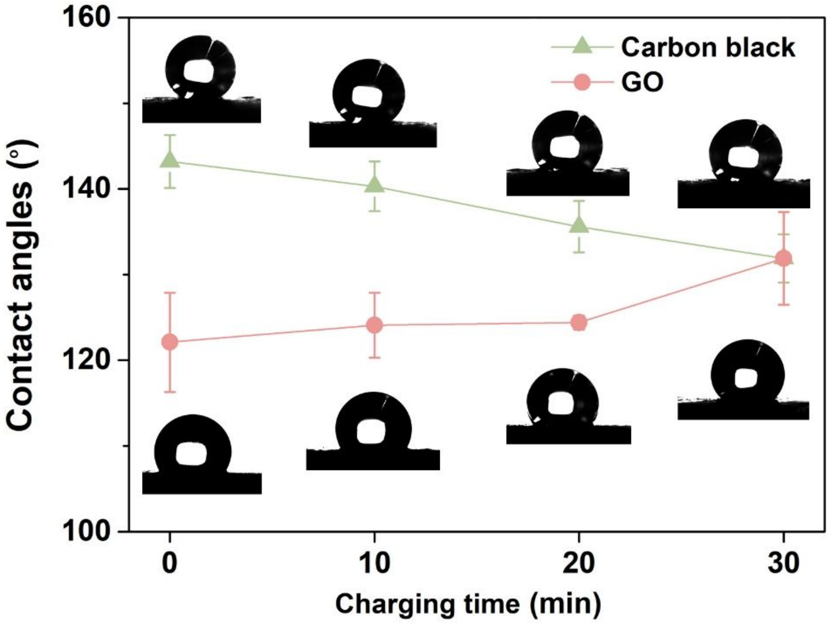

Figure S-3 Water in air contact angle change of GO on glassy carbon and carbon black on glassy carbon as a function of time exposed to electrochemical conditioning at -1.2 V vs Ag/AgCl in 0.1 M $KHCO_3$. We measured the contact angles of rinsed electrodes by the Sessil drop method. The error bars on contact angle data points represent the standard deviation of nine measurements on three areas of three independently prepared samples.

Figure S-3 compares the change in the wetting contact angle of the GO thin film treated for different lengths of time by electrochemical conditioning at -1.2 V versus Ag|AgCl to the change in the wetting contact angle of a carbon black control sample. Initially, the carbon black sample is more hydrophobic (contact angle = 143.2 ± 3.1°) than the GO.

However, the carbon black loses its hydrophobicity after the contact angle drops to 131.9±2.8° after 30 minutes of cathodic treatment. In contrast, as more of the GO surface sites become reduced to r-GO with increased treatment time, the GO sample becomes more hydrophobic with the contact angle increasing from 122.1 ± 5.8° to 131.9±5.4° after 30 minutes of treatment. The shift to higher contact angles and greater hydrophobicity we observed is consistent with trends reported for bulk GO films by Feng et al.[4] and GO reduced on steel mesh by Lu et al. [5]. The improved hydrophobicity of the GO thin film may owe to the reduction of oxygen functional groups on the GO surface. Oxygen functional groups generally promote wetting of carbon surfaces by enhancing dipole-dipole interactions with the water molecules.[6] It is noted that the declined range is not as large as expected. The surface contact angle is decided by more than the chemical composition. Other factors such as surface microstructure and hierarchical structure contribute to the change as well.

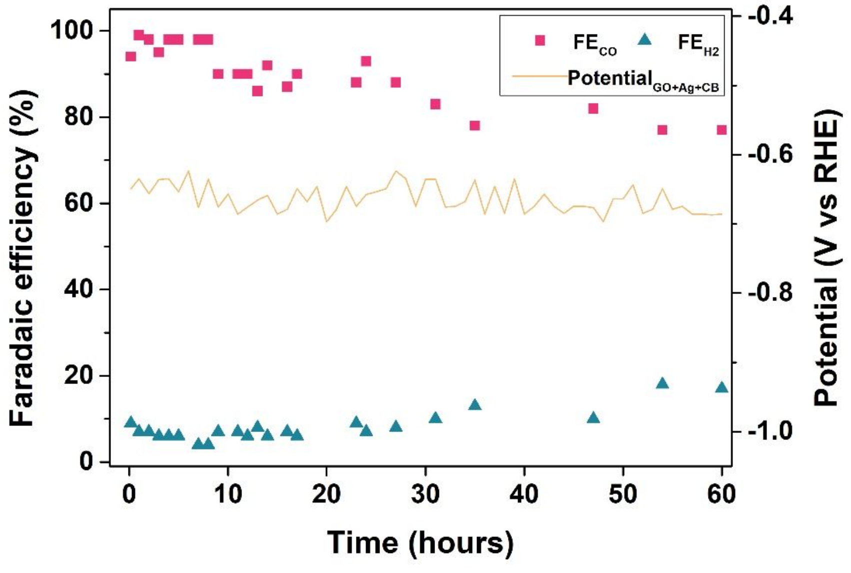

Figure S-4 Faradaic efficiency for CO and H_2 and cathode potential for the GO+Ag+CB GDE during an additional 60 h stability test at a current density of 100 mA·cm^{-2}

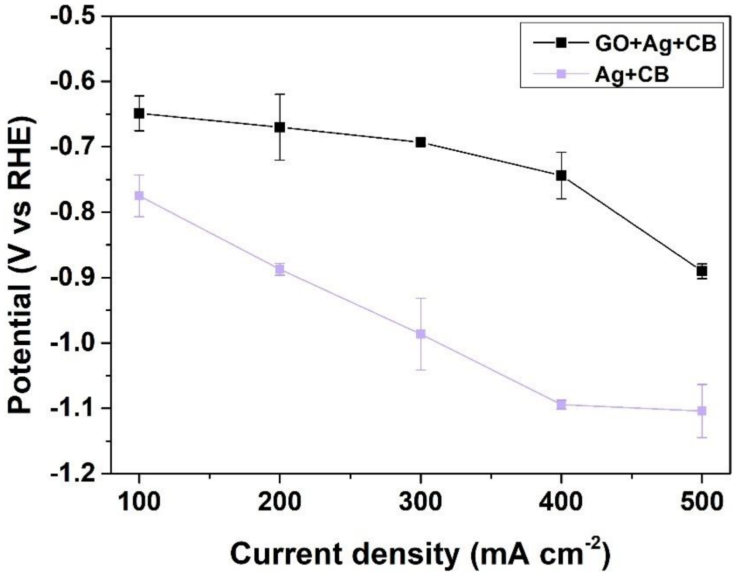

Figure S-5 Cathode potentials during the CO_2 electrolysis measurement at a chronopotentiometry mode

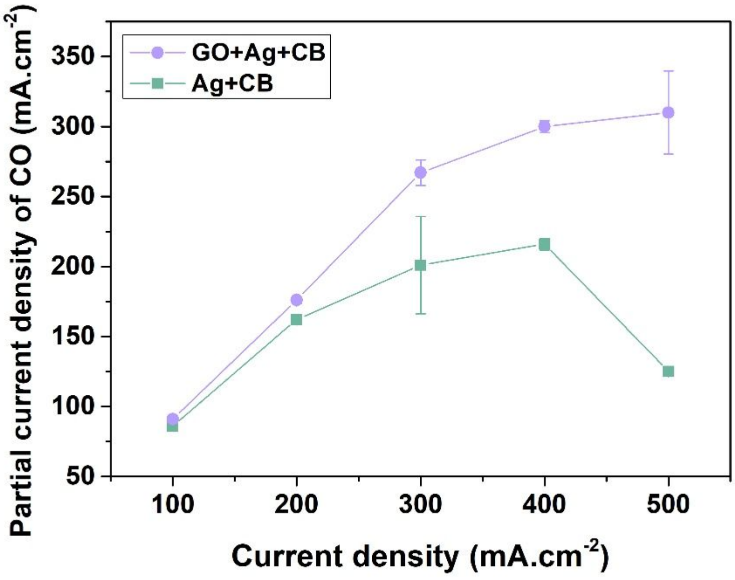

Figure S-6 Partial current densities of carbon monoxide during CO_2 electrolysis on Ag+CB and GO+Ag+CB GDEs respectively

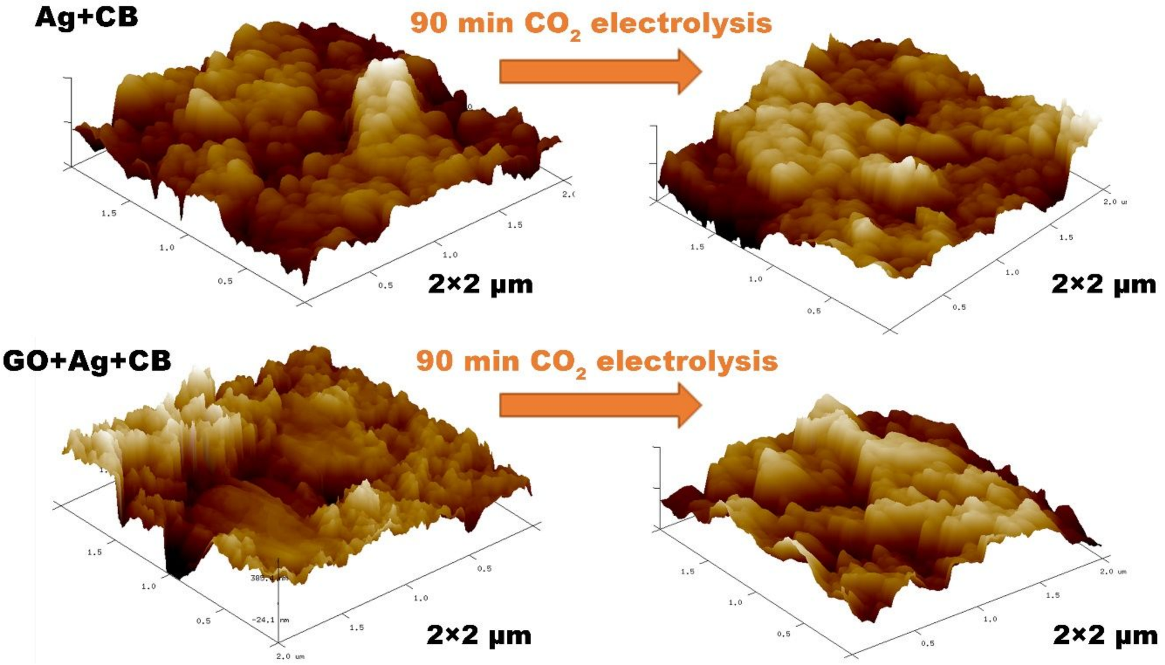

Figure S-7 AFM topography of Ag+CB GDE (above) and GO+Ag+CB GDE (below) before and after the CO_2 electrolysis measurements

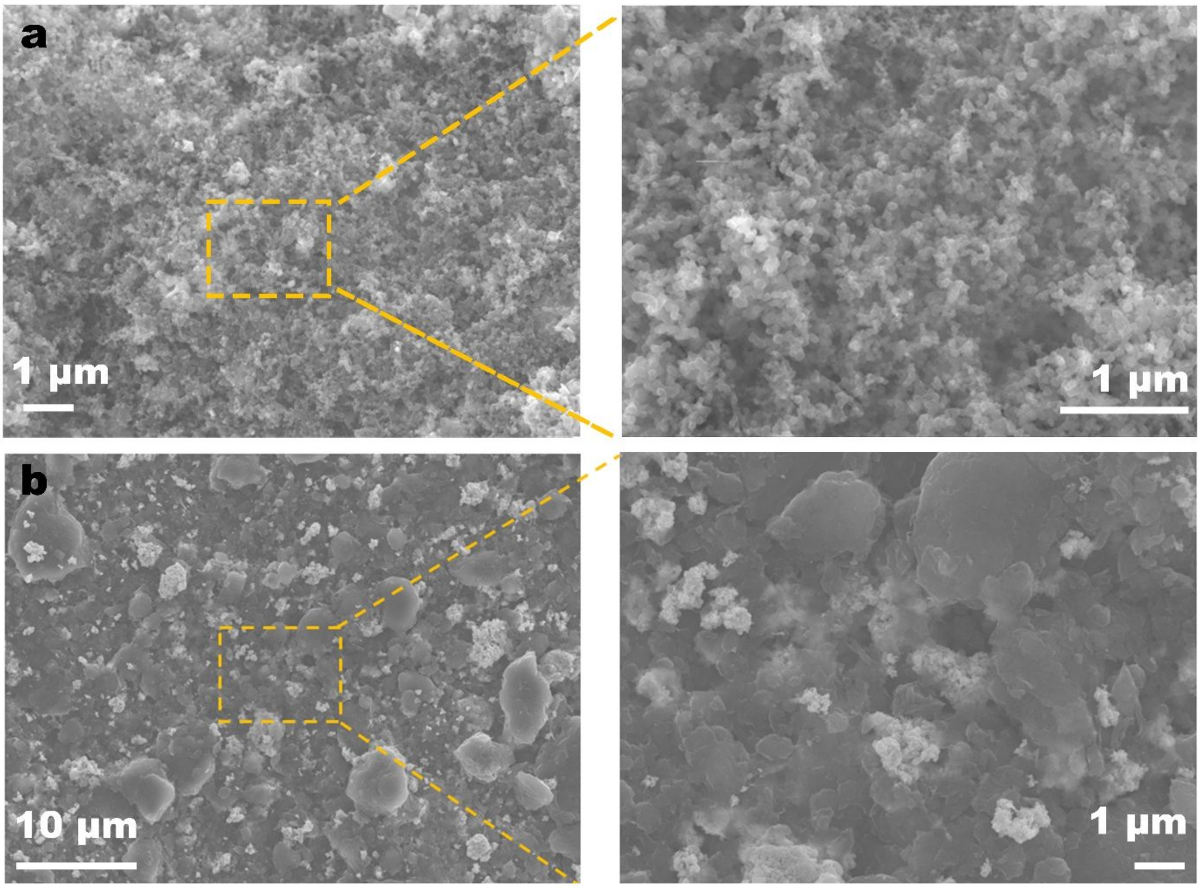

Figure S-8 SEM images at different magnifications of fresh catalyst layer on (a) Ag+CB GDE and (b) GO+Ag GDE

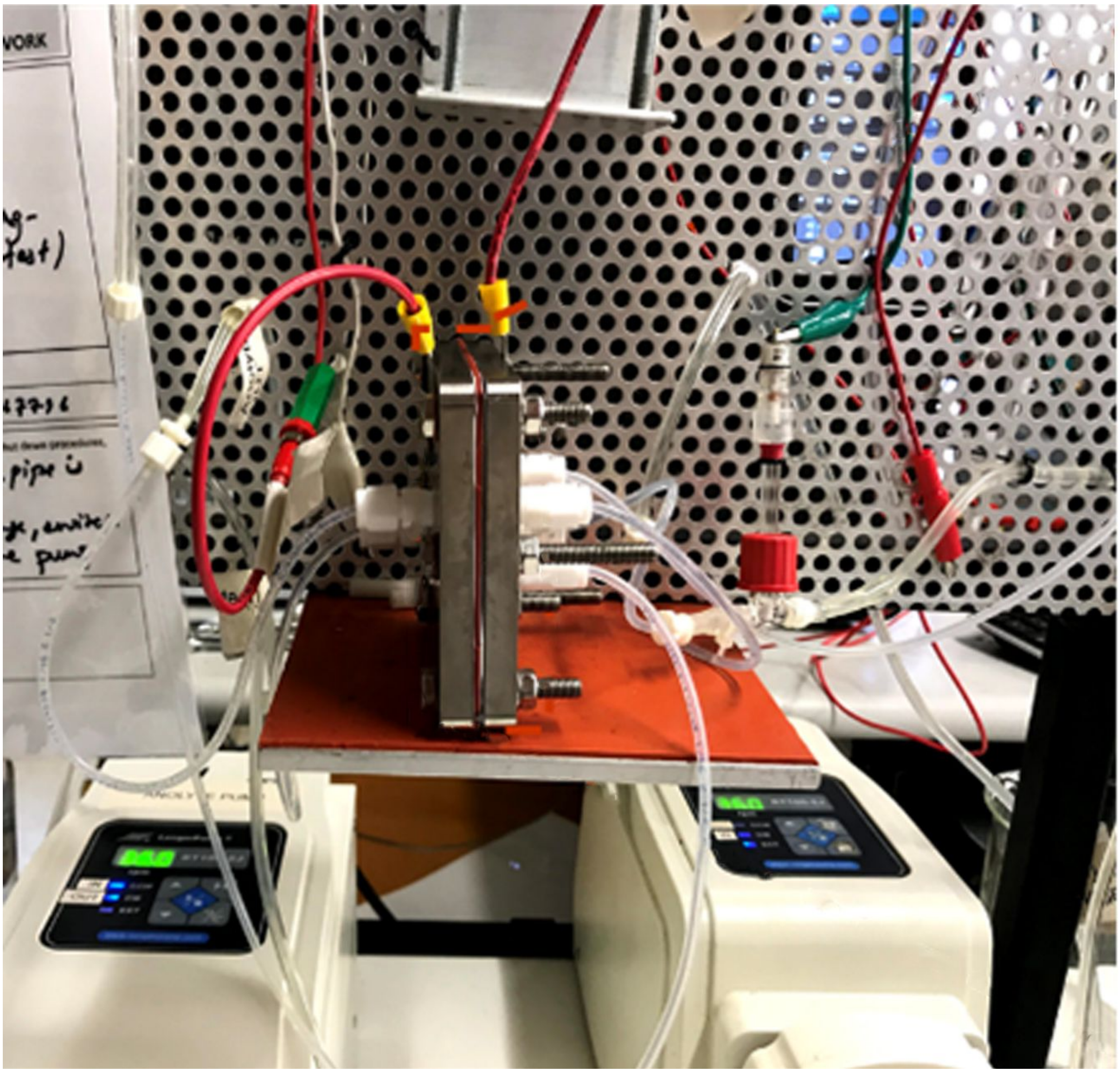

Figure S-9 Picture of our custom-made flow cell electrolyzer and system for CO_2 electrolysis

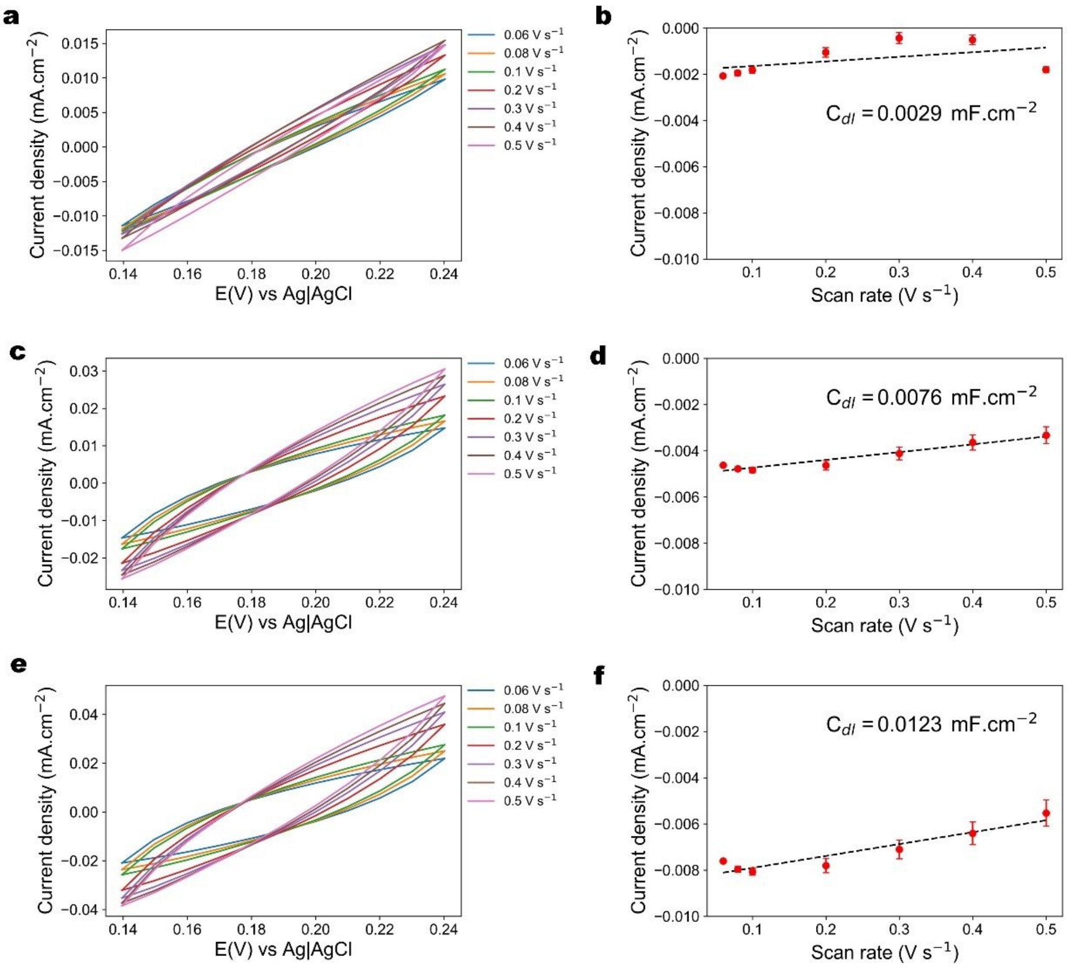

Figure S-10 CV curves at 7 scan rates of Ag+CB GDEs after (a) 0 min fresh, (c) 60 min and (e) 90 min CO_2 electrolysis measurements; Corresponding charging current density vs scan rate of Ag+CB GDE after (b) 0 min fresh, (d) 60 min, and (f) 90 min CO_2 electrolysis measurements

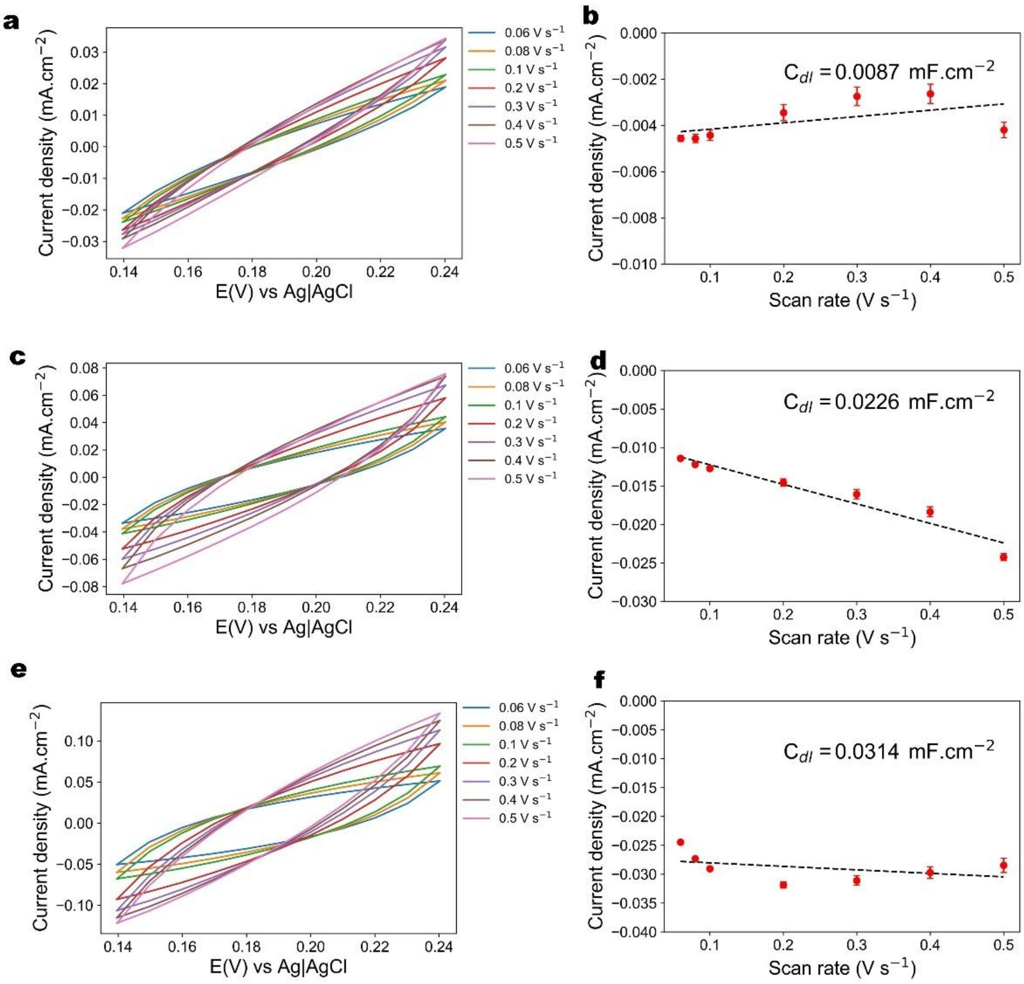

Figure S-11 CV curves at 7 scan rates of GO+Ag+CB GDEs after (a) 0 min fresh, (c) 60 min and (e) 90 min CO2RR measurements; Corresponding charging current density vs scan rate of GO+Ag+CB GDE after (b) 0 min fresh, (d) 60 min, and (f) 90 min CO_2 electrolysis measurements

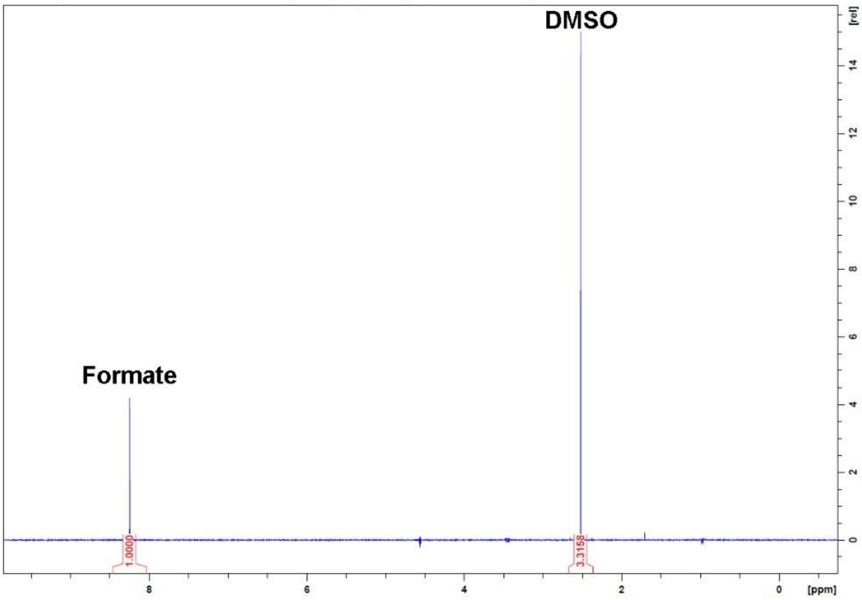

Figure S-12 A representative NMR spectrum of collected effluent catholyte from CO_2 electrolysis at 500 mA.cm^{-2} for 10 minutes

References

1. G. K. Ramesha and S. Sampath, *The Journal of Physical Chemistry C*, 2009, **113**, 7985-7989.
2. Y. Shao, J. Wang, M. Engelhard, C. Wang and Y. Lin, *Journal of Materials Chemistry*, 2010, **20**, 743-748.
3. J.-B. Wu, M.-L. Lin, X. Cong, H.-N. Liu and P.-H. Tan, *Chemical Society Reviews*, 2018, **47**, 1822-1873.
4. X. Feng, W. Chen and L. Yan, *RSC Advances*, 2016, **6**, 80106-80113.
5. H. Lu, S. Sha, S. Yang, J. Wu, J. Ma, C. Hou and Z. Sheng, *Applied Surface Science*, 2021, **538**, 147948.
6. M. Li, M. N. Idros, Y. Wu, T. Burdyny, S. Garg, X. S. Zhao, G. Wang and T. E. Rufford, *Journal of Materials Chemistry A*, 2021, **9**, 19369-19409.
